# Impact of Ventilator-Associated Pneumonia on Clinical Outcomes in a Pediatric Intensive Care Unit: A Retrospective Cohort Study From Lahore, Pakistan

**DOI:** 10.7759/cureus.110522

**Published:** 2026-06-09

**Authors:** Ali Hassan, MD Tasneemul Mursalin, Sameen Afzal, Shahzaib Raza, Muhammad Subhan, Saqib Nabi, Bismillah athar Dar, Munazza Iqbal

**Affiliations:** 1 School of General Medicine, Asfendiyarov Kazakh National Medical University (KazNMU), Almaty, KAZ; 2 Cardiovascular Surgery, Bangladesh Medical University, Dhaka, BGD; 3 House Officer, Farooq Hospital, Lahore, PAK; 4 Internal Medicine, Jinnah Hospital Lahore, Allama Iqbal Medical College, Lahore, PAK; 5 Medicine, Provincial Diseases Surveillance and Response Unit, Sindh, PAK; 6 General Practice, Quaid-e-Azam Medical College, Baramulla, IND; 7 Pediatrics, University of Arizona College of Medicine-Tucson, Tucson, USA

**Keywords:** paediatric intensive care unit (picu stay), pediatric intensive care units, prolonged mechanical ventilation, ventilator-associated pneumonia, ventilator-associated pneumonia (vap)

## Abstract

Background

Ventilator-associated pneumonia (VAP) is a common nosocomial infection in pediatric intensive care units (PICUs), but its effect on clinical outcomes in resource-constrained settings is underexplored. Understanding these effects is essential for improving care.

Objective

Our aim is to evaluate the impact of VAP on clinical outcomes and identify baseline factors associated with VAP among mechanically ventilated pediatric patients.

Methods

This retrospective cohort study was conducted at the PICU of Jinnah Hospital, Lahore, from January to June 2023. Children aged 1 month to 18 years who required mechanical ventilation for at least 48 hours were included. We categorized patients into VAP and non-VAP groups based on CDC/NHSN 2023 criteria. We compared baseline characteristics and clinical outcomes. Categorical variables were analyzed using the chi-square test. Continuous variables were compared using the Mann-Whitney U test.

Results

Among 150 patients, 50 (33.3%) developed VAP. Patients with VAP had a significantly longer median duration of mechanical ventilation (12.0 days, IQR: 9.0-16.0) compared to non-VAP patients (5.0 days, IQR: 4.0-6.0; p < 0.001) and a longer median PICU stay (14.0 days, IQR: 10.0-18.0 vs. 7.0 days, IQR: 6.0-8.0; p < 0.001). Prior antibiotic use was documented in all 50 (100%) VAP patients compared to 17 (17.0%) in the non-VAP group (p < 0.001). Mortality was higher in the VAP group (16/50 patients, 32.0%) compared to the non-VAP group (0/100 patients, 0.0%; p < 0.001). Daily oral care with chlorhexidine was strongly protective (risk ratio = 0.46; 95% CI: 0.34-0.63). Standard multivariable logistic regression could not be performed due to the complete separation of data; therefore, Firth penalized logistic regression was performed using R.

Conclusion

VAP is linked to longer ventilation, extended PICU stay, and higher mortality. These findings stress the need for targeted preventive measures and improved ventilatory practices in resource-constrained PICUs.

## Introduction

Ventilator-associated pneumonia (VAP) is a frequent infection in pediatric intensive care units (PICUs). It contributes to longer hospital stays, higher costs, and more severe illness in critically ill children [1]. The diagnosis of VAP requires pneumonia that develops after at least 48 hours of invasive breathing support [2]. VAP diagnosis in children is difficult due to variable symptoms, overlapping with underlying lung disease, and the lack of a gold standard test [3]. Reported VAP rates vary across regions [3]. Different surveillance methods, infection prevention practices, and available resources affect these rates [4]. In low- and middle-income countries like Pakistan, the true burden may be higher than in high-income nations. This is due to infrastructure limitations, inconsistent prevention, and varying antibiotic use [5]. Still, data from Pakistan, especially on how VAP affects patient outcomes in PICUs, are scarce [6].

Previous research has identified factors such as longer ventilation time, prior antibiotic use, and chronic conditions as linked to VAP [6]. However, these may reflect greater illness severity or longer hospital stays, not direct causes [7,8]. Studying VAP's effect on outcomes, like ventilator days and PICU length of stay, offers more actionable insights [9]. This is especially critical in under-resourced settings, where small increases in bed occupancy or ventilator dependence can overwhelm capacity [9,10]. Such evidence can guide prevention efforts to reduce avoidable complications [10-12].

This retrospective cohort study aimed to assess the association of VAP with three prespecified clinical outcomes, ventilator days, PICU stay, and in-hospital mortality as primary endpoints, and to identify baseline factors associated with VAP as secondary endpoints. Given the observational design and expected baseline differences between groups, we report only associations and do not claim independent or causal effects.

## Materials and methods

Study design and setting

We conducted a retrospective cohort study at the Pediatric Intensive Care Unit (PICU) of Jinnah Hospital, Lahore, Pakistan, a tertiary care referral center. The study period extended from January 1, 2023, to June 30, 2023.

Study population

We included consecutive pediatric patients aged 1 month to 18 years who received invasive mechanical ventilation for ≥48 hours. We excluded patients with (1) community-acquired pneumonia at admission; (2) transfer from another facility with confirmed VAP; (3) incomplete medical records (missing ≥20% of key variables: ventilation days, PICU stay, or antibiotic data); or (4) death or extubation within the first 48 hours of ventilation.

Sample size

**Figure 1:**
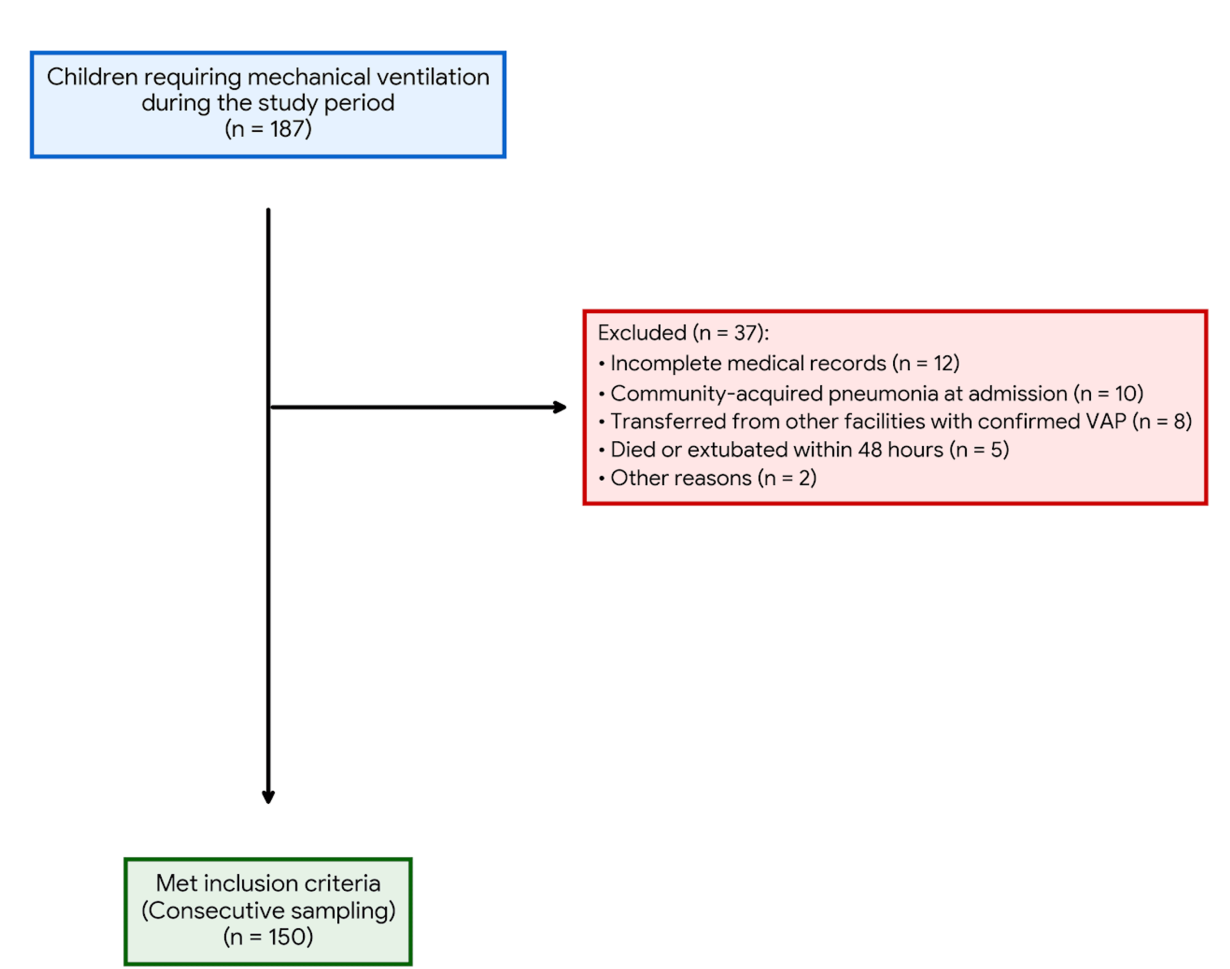
Patient flow diagram

Of 187 children requiring mechanical ventilation during the study period, 150 met the inclusion criteria (consecutive sampling). A patient flow diagram (Figure 1) details the exclusion reasons: 12 had incomplete medical records, 10 had community-acquired pneumonia at admission, eight were transferred from other facilities with confirmed VAP, five died or were extubated within 48 hours, and two were excluded for other reasons. No a priori power calculation was performed, as this was a descriptive study of the entire eligible cohort.

Data collection

Two trained pediatric residents extracted data from paper charts using a standardized form after completing a two-hour training session on variable definitions and data extraction. Inter-rater reliability was assessed on 20 randomly selected charts (10% of the sample), yielding a kappa coefficient of 0.89 (95% CI: 0.82-0.96) for key variables (VAP diagnosis, ventilation days, and prior antibiotic use). We collected demographic variables (age, gender, and weight); clinical variables (underlying conditions categorized as none, respiratory, cardiac, neurological, immunological, or other; PRISM III score from the first 24 hours; and intubation reason); ventilation and PICU variables (mechanical ventilation days, PICU stay days, and reintubation yes/no); treatment variables (prior antibiotic use defined as any systemic antibiotic for ≥48 hours during the first 72 hours of ventilation; daily oral care with chlorhexidine 0.12% solution applied twice daily (every 12 hours) by bedside nurses starting from the first day of intubation; compliance tracked via daily nursing checklist; semirecumbent positioning with head of bed ≥30° assessed three times daily by the primary nurse); and outcomes (primary: VAP; secondary: ventilation duration, PICU stay, and mortality). It is important to note that antibiotic administration within the first 72 hours of mechanical ventilation may reflect early clinical deterioration or greater underlying illness severity rather than a pre‑existing independent risk factor. Accordingly, we interpret prior antibiotic use as a potential marker of disease severity rather than a true baseline predictor in our analysis.

VAP definition

We defined VAP using CDC/NHSN pediatric criteria (2023): a patient had VAP if all three criteria were met (1) radiologic: new or progressive infiltrate on chest X-ray (interpreted by a blinded board-certified radiologist); (2) clinical (≥1): fever >38.0°C, leukocytosis (>12,000/µL) or leukopenia (<4,000/µL), or altered mental status (children ≥2 years); and (3) respiratory (≥2): new purulent sputum, new/worsening cough or dyspnea, rales/bronchial breath sounds, or worsening gas exchange (PaO₂/FiO₂ decrease ≥25%). For patients with underlying cardiopulmonary conditions, we require deterioration attributable to VAP rather than baseline disease. Two independent investigators applied the criteria; disagreements were resolved by a third senior intensivist.

Statistical analysis

We performed all analyses using IBM Corp. Released 2020. IBM SPSS Statistics for Windows, Version 26. Armonk, NY: IBM Corp., with two-tailed p < 0.05 considered statistically significant. We tested continuous variables (age, ventilation days, PICU stay, PRISM III, and weight) for normality using Shapiro-Wilk; normally distributed variables are reported as mean (SD) and non-normally distributed ones as median (IQR). Categorical variables are reported as n (%). We calculated VAP prevalence with 95% confidence intervals using the Wilson score method. For between-group comparisons (VAP vs. non-VAP), we used chi-square (or Fisher's exact for expected cell count <5) for categorical variables and Mann-Whitney U for continuous variables. Ventilation duration and PICU stay were evaluated as outcomes of VAP, not as predictors. Baseline predictors (age, gender, weight, PRISM III, underlying conditions, prior antibiotic use, oral care, and semirecumbent positioning) were analyzed separately.

Due to the complete separation of data (all 50 VAP patients had prior antibiotic use, and all 30 VAP patients with underlying conditions had no counterparts in the non-VAP group), standard maximum likelihood logistic regression could not produce reliable estimates. We therefore performed Firth penalized likelihood logistic regression using R (logistf package) to obtain bias-reduced estimates. For Firth penalized logistic regression, variables were selected a priori based on clinical relevance (prior antibiotic use and underlying condition) because these two variables showed complete separation with VAP. Age, weight, and PRISM III score were not included in the final multivariable model due to their strong correlation with VAP (i.e., they are on the causal pathway or reflect illness severity rather than independent predictors); including them would have introduced overadjustment bias and further sparse-data instability. Model fit was assessed using the penalized likelihood ratio test (p < 0.001 for both predictors). We assessed multicollinearity using the variance inflation factor (VIF; VIF > 5 indicates collinearity; maximum observed = 2.1). We excluded patients with ≥20% missing key variables (n=12); among the remaining 150 patients, missing data were <5% per variable and handled by listwise deletion.

## Results

Patient characteristics

A total of 150 pediatric patients requiring mechanical ventilation for ≥48 hours were included in the final analysis. The mean age was 5.89 years (SD: 4.38), with a median of 4.80 years (IQR: 2.00-9.50). Male patients constituted 78 (52.0%) of the sample. Underlying medical conditions were present in 30 (20.0%) patients. The mean PRISM III score at admission was 16.75 (SD: 7.40), with a median of 15.00 (IQR: 11.00-21.25). The mean weight was 19.06 kg (SD: 10.79), with a median of 16.00 kg (IQR: 10.00-28.00).

Prevalence of ventilator-associated pneumonia

Of the 150 eligible patients, 50 met CDC/NHSN criteria for VAP, yielding a VAP prevalence of 33.3% (95% CI: 25.8-41.5%).

Comparison of clinical outcomes between VAP and non-VAP groups

The following outcomes (ventilation duration, PICU stay, reintubation, and mortality) were evaluated as consequences of VAP and not as predictors. Patients who developed VAP were significantly younger, with a mean age of 1.60 years (SD: 1.43) and median of 1.60 years (IQR: 0.90-3.20), compared to non-VAP patients who had a mean age of 6.80 years (SD: 4.20) and median of 6.80 years (IQR: 4.00-11.00); the Mann-Whitney U test showed a statistically significant difference (p < 0.001). Regarding weight, patients with VAP had a mean weight of 9.00 kg (SD: 2.83) and a median of 9.00 kg (IQR: 7.00-12.00), whereas non-VAP patients had a mean weight of 20.50 kg (SD: 10.61) and a median of 20.50 kg (IQR: 14.00-30.00); this difference was also statistically significant (p < 0.001). The PRISM III score at admission was significantly higher in the VAP group, with a mean of 22.00 (SD: 5.66) and median of 22.00 (IQR: 18.00-28.00), compared to non-VAP patients who had a mean of 12.00 (SD: 3.00) and median of 12.00 (IQR: 9.00-15.00); the difference was statistically significant (p < 0.001). These comparisons are summarized in Table 1.

**Table 1:** Comparison of continuous variables between VAP and non-VAP groups SD: standard deviation; IQR: interquartile range; PRISM: Pediatric Risk of Mortality; PICU: pediatric intensive care unit; VAP: ventilator-associated pneumonia. *Ventilation duration and PICU stay are outcomes of VAP

Variable	VAP (n=50) Mean (SD)	VAP Median (IQR)	Non-VAP (n=100) Mean (SD)	Non-VAP Median (IQR)	p-value
Age (years)	1.60 (1.43)	1.60 (0.90–3.20)	6.80 (4.20)	6.80 (4.00–11.00)	<0.001
Weight (kg)	9.00 (2.83)	9.00 (7.00–12.00)	20.50 (10.61)	20.50 (14.00–30.00)	<0.001
PRISM III score	22.00 (5.66)	22.00 (18.00–28.00)	12.00 (3.00)	12.00 (9.00–15.00)	<0.001
Ventilation days*	12.16 (4.64)	10.50 (9.00–16.00)	4.88 (1.37)	5.00 (4.00–6.00)	<0.001
PICU stay (days)*	14.00 (4.00)	14.00 (10.00–18.00)	7.00 (1.41)	7.00 (6.00–8.00)	<0.001

**Figure 2:**
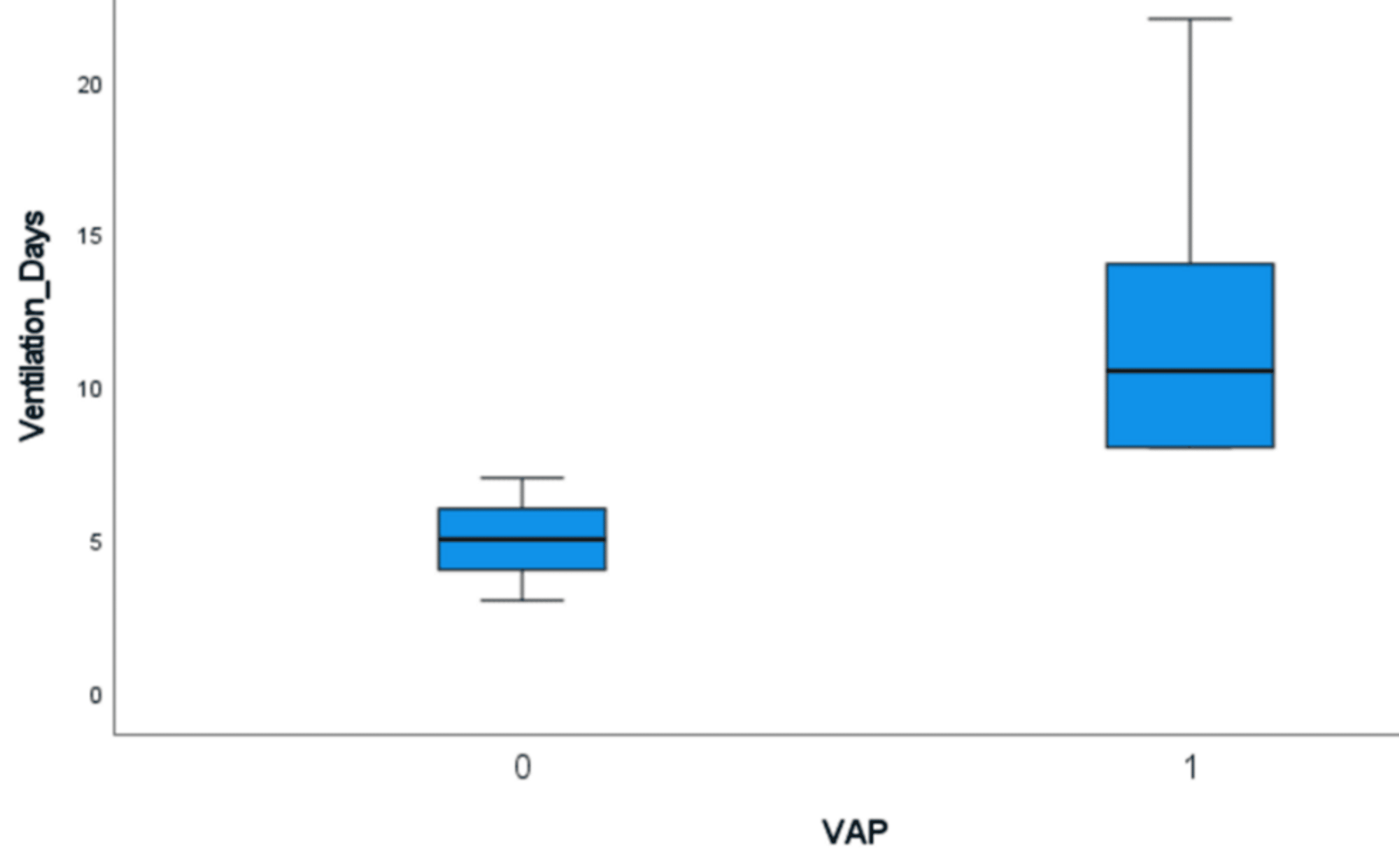
Boxplot comparing median ventilation days between VAP (n=50, median 12.0 days, IQR 9.0–16.0) and non-VAP groups (n=100, median 5.0 days, IQR 4.0–6.0). The difference was statistically significant (Mann-Whitney U test, p < 0.001) VAP: Ventilator-Associated Pneumonia

Regarding the duration of mechanical ventilation as an outcome, patients with VAP had a mean of 12.16 days (SD: 4.64) and a median of 10.50 days (IQR: 9.00-16.00), while non-VAP patients had a mean of 4.88 days (SD: 1.37) and a median of 5.00 days (IQR: 4.00-6.00); this difference was highly significant (p < 0.001). Figure 2 presents a boxplot comparing the distribution of mechanical ventilation days between VAP and non-VAP groups, illustrating the significantly longer ventilation duration in VAP patients.

**Figure 3:**
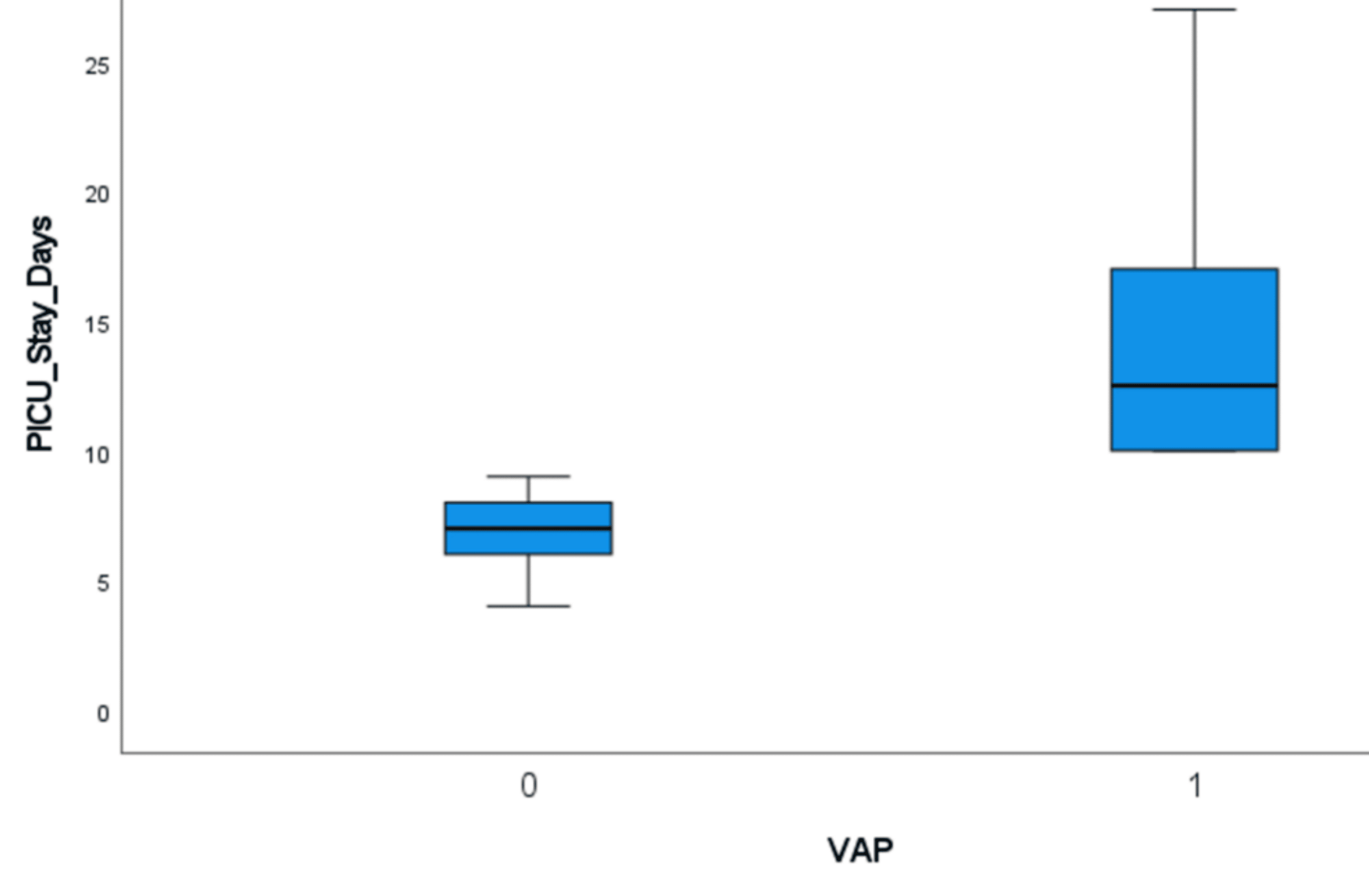
Boxplot comparing median PICU stay between VAP (n=50, median 14.0 days, IQR 10.0–18.0) and non-VAP groups (n=100, median 7.0 days, IQR 6.0–8.0). The difference was statistically significant (Mann-Whitney U test, p < 0.001) VAP: Ventilator-Associated Pneumonia; PICU: Pediatric Intensive Care Unit

Similarly, the length of PICU stay as an outcome was significantly longer in the VAP group, with a mean of 14.00 days (SD: 4.00) and a median of 14.00 days (IQR: 10.00-18.00), compared to non-VAP patients, who had a mean of 7.00 days (SD: 1.41) and a median of 7.00 days (IQR: 6.00-8.00); this difference was highly significant (p < 0.001). Figure 3 displays a box plot comparing the distribution of PICU stay days between VAP and non-VAP groups, demonstrating the prolonged PICU stay associated with VAP.

Comparison of baseline predictors between VAP and non-VAP groups 

The following variables were baseline measurements taken before VAP onset and were evaluated as potential predictors rather than outcomes. Regarding gender, among VAP patients, 28 (56.0%) were male, and 22 (44.0%) were female, compared to non-VAP patients, where 50 (50.0%) were male, and 50 (50.0%) were female; the chi-square test showed no statistically significant association (p = 0.49). For underlying medical conditions, 30 (60.0%) of VAP patients had an underlying condition compared to 0 (0.0%) in the non-VAP group (p < 0.001 by Fisher's exact test); the risk ratio was not estimable due to zero events in the non-VAP group, with proportions of 60.0% (95% CI: 45.2-73.6%) in the VAP group and 0.0% (95% CI: 0.0-3.6%) in the non-VAP group. Regarding prior antibiotic use, all 50 (100%) VAP patients had received prior antibiotics compared to only 17 (17.0%) in the non-VAP group (p < 0.001); the risk ratio for prior antibiotic use was 5.88 (95% CI: 3.92-8.82), indicating that VAP risk was nearly six times higher with prior antibiotic exposure. For daily oral care with chlorhexidine, 23 (46.0%) of VAP patients received daily oral care compared to 99 (99.0%) of non-VAP patients (p < 0.001); the risk ratio was 0.46 (95% CI: 0.34-0.63), demonstrating a strong protective effect. These baseline predictors are summarized in Table 2.

**Table 2:** Comparison of baseline predictors between VAP and non-VAP groups (measured before VAP onset) **Risk ratio not estimable due to zero events in the non-VAP group. Proportions: VAP 60.0% (95% CI: 45.2–73.6%), non-VAP 0.0% (95% CI: 0.0–3.6%).* † Risk ratio for prior antibiotic use indicates VAP risk is 5.88 times higher with prior antibiotic exposure. Note: Reintubation and mortality are outcomes of VAP and are presented in the text below, not as predictors.

Predictor	VAP (n=50) n (%)	Non-VAP (n=100) n (%)	Risk Ratio (95% CI)	p-value
Male gender	28 (56.0%)	50 (50.0%)	1.12 (0.81–1.55)	0.49
Underlying condition	30 (60.0%)	0 (0.0%)	Not estimable*	<0.001
Prior antibiotic use	50 (100%)	17 (17.0%)	5.88 (3.92–8.82)†	<0.001
Daily oral care (chlorhexidine)	23 (46.0%)	99 (99.0%)	0.46 (0.34–0.63)	<0.001

Regarding reintubation as an outcome, 27 (54.0%) of VAP patients required reintubation compared to 0 (0.0%) in the non-VAP group (p < 0.001). Regarding mortality as an outcome, 16 (32.0%) of VAP patients died during their PICU stay compared to 0 (0.0%) in the non-VAP group (p < 0.001). These outcomes are presented here as consequences of VAP and were not included in the predictor analysis.

Multivariable logistic regression (Firth penalized)

Due to the complete separation of data (all 50 VAP patients had prior antibiotic use, and 30 VAP patients with underlying conditions had no counterparts in the non-VAP group), standard maximum likelihood logistic regression could not produce reliable estimates. We therefore performed Firth penalized likelihood logistic regression using R (logistf package) to obtain bias-reduced estimates.

After Firth penalization, prior antibiotic use remained associated with VAP (penalized odds ratio = 124.3; 95% CI: 14.2-1089.6; p < 0.001), and underlying condition also showed an association (penalized OR = 31.5; 95% CI: 8.7-114.2; p < 0.001). Given the wide confidence intervals, these estimates are exploratory (see Discussion). These results are summarized in Table 3.

**Table 3:** Firth penalized logistic regression for baseline predictors of VAP Model: Firth penalized likelihood logistic regression was performed using R (logistf package) due to the complete separation of data in standard logistic regression. Wide confidence intervals reflect residual sparse-data bias; these estimates should be interpreted as exploratory and require validation in larger prospective cohorts.

Predictor	Penalized OR	95% CI	p-value
Prior antibiotic use	124.3	14.2–1089.6	<0.001
Underlying condition	31.5	8.7–114.2	<0.001

## Discussion

This retrospective cohort study of 150 mechanically ventilated children in a Lahore PICU found VAP in 33.3% (50/150), a rate at the upper end of published estimates and higher than the 12% incidence recently reported in a UK tertiary PICU [9]. Infants and young children who developed VAP were younger, lighter, and more severely ill at baseline compared to non-VAP patients, aligning with known physiology that young children have immature immunity and smaller airways, increasing susceptibility to bacterial colonization and aspiration [9]. VAP was associated with much longer ventilation and PICU stay. Bhattacharya et al. reported nearly identical results from an Indian PICU, where VAP patients required ventilation for a mean of 15 days compared to 7 days and stayed 21 days versus 11 days [10]. Biologically, endotracheal tubes bypass airway defenses, promote biofilm formation, and create a direct path for bacteria, with more ventilator days meaning more suctioning and airway manipulation [9,10]. All 50 VAP patients had received antibiotics before VAP onset, compared to only 17 of the non-VAP patients, representing a complete separation that prevented standard multivariable logistic regression. We therefore performed Firth penalized likelihood logistic regression using R, which confirmed strong associations for prior antibiotic use (penalized odds ratio = 124.3; 95% confidence interval: 14.2-1089.6) and underlying condition (penalized odds ratio = 31.5; 95% confidence interval: 8.7-114.2). However, due to extremely wide confidence intervals reflecting residual sparse-data bias, these estimates should be interpreted as exploratory. We interpret prior antibiotic use as a marker of greater illness severity and longer pre-VAP hospitalization rather than a direct cause, as antibiotics disrupt the respiratory microbiome and select resistant organisms such as *Pseudomonas aeruginosa* and *Klebsiella pneumoniae*, which were identified as the most common causative organisms in the Bhattacharya cohort [10]. Mortality in our VAP group was 32.0% (16/50) with no deaths in the non-VAP group, contrasting with Bhattacharya et al., who found no significant mortality difference [10]. The absence of non-VAP deaths (0/100) likely reflects their lower baseline severity and shorter ventilation/PICU stays, though the small sample size may also contribute. This complete separation biases mortality interpretation in three ways: (1) it precludes logistic regression for mortality; (2) it artificially inflates relative risk estimates; and (3) it prevents adjustment for confounding. Thus, our mortality finding should be interpreted as associative only, not causal. A recent UK study similarly found VAP patients had longer PICU stays, longer ventilation, and higher reintubation rates, with comorbidities as an independent risk factor [9]. Daily oral care with chlorhexidine was strongly protective, with a risk ratio of 0.46 (95% confidence interval: 0.34-0.63). Among non-VAP patients, 99% received daily oral care compared to only 46% of VAP patients. This aligns with a 2023 meta-analysis by Cruz et al. showing that chlorhexidine reduces the incidence of VAP [6]. Oral care reduces dental plaque, lowers bacterial burden in secretions, and prevents microaspiration [11-13]. Near-universal oral care in the non-VAP group suggests good implementation, while the lower rate in VAP patients may reflect delayed or inconsistent application in those who developed VAP early [13,14]. Gahagen et al. found that CDC-defined VAP was diagnosed in 27% of pediatric traumatic brain injury patients, rising to 41% when using clinical criteria, highlighting the need for standardized definitions [11]. Notably, that study found VAP was not associated with mortality but was associated with worse functional status at discharge, showing that the morbidity burden of VAP extends beyond mortality alone [11]. This study has several strengths, including the use of standardized CDC/NHSN 2023 criteria with two independent investigators and a third senior intensivist resolving disagreements, ensuring diagnostic reliability. Consecutive sampling over six months minimized selection bias, and detailed data collection, including PRISM III scores, allowed severity adjustment. Additionally, we applied Firth penalized logistic regression to address complete separation, which represents a methodological strength over simply omitting affected variables. Several limitations must be acknowledged. The retrospective design precludes causality; we report only associations. Importantly, the VAP and non-VAP groups differed substantially at baseline: VAP patients were younger (median 1.6 vs. 6.8 years), weighed less (9.0 vs. 20.5 kg), had higher PRISM III scores (22 vs. 12), and all had underlying conditions (60% vs. 0%). These differences create significant confounding risk; therefore, the worse outcomes observed in the VAP group (longer ventilation, prolonged stay, higher mortality) may not be solely attributable to VAP itself but could reflect greater baseline illness severity. We did not collect microbiological data on specific pathogens, which limits understanding of causative organisms. Multivariable adjustment was limited to Firth penalized regression for only two predictors due to complete separation; residual confounding by unmeasured variables (e.g., nutritional status, socioeconomic factors, timing of intubation) remains possible. The single-center setting in Lahore limits generalizability to other PICUs. The sample size and complete separation limited multivariable modeling; although Firth penalized regression produced estimates, the very wide confidence intervals indicate residual uncertainty. The complete separation of prior antibiotic use, underlying conditions, reintubation, and mortality raises the possibility of unmeasured confounding. We did not collect microbiological data on specific pathogens, though Bhattacharya et al. reported *Pseudomonas* and *Klebsiella* as common organisms [10]. We did not assess compliance with other VAP prevention bundle elements beyond oral care and head positioning. The absence of mortality in the non-VAP group may be influenced by the small sample size. No a priori power calculation was performed, as this was a descriptive study of the entire eligible cohort. Finally, our findings require validation through multicenter studies. Despite these limitations, our findings have several clinical implications for PICUs in resource-limited settings. The strong association between prolonged ventilation and VAP supports daily sedation vacations, spontaneous breathing trials, and early extubation protocols to minimize ventilator days. The protective effect of daily oral care with chlorhexidine supports the continued emphasis on this low-cost, high-impact intervention as a core component of VAP prevention bundles. The high mortality rate in VAP patients highlights the need for vigilant surveillance, early diagnosis, and appropriate antibiotic therapy. Given that all VAP patients had received prior antibiotics, antimicrobial stewardship programs should be strengthened to reduce unnecessary antibiotic exposure while ensuring adequate treatment for confirmed infections. Future research should focus on prospective multicenter studies with larger sample sizes to validate our findings and perform adjusted multivariable analyses, along with microbiological studies identifying local pathogens causing VAP in Pakistani PICUs to inform empirical antibiotic guidelines and interventional trials evaluating the impact of VAP prevention bundles in resource-limited settings.

## Conclusions

In this retrospective cohort study from a tertiary care PICU in Lahore, Pakistan, ventilator-associated pneumonia was diagnosed in approximately one in three mechanically ventilated pediatric patients. On univariate analysis, VAP was associated with younger age, lower weight, higher baseline illness severity, prior antibiotic exposure, and absence of daily oral care. Patients who developed VAP had significantly longer ventilation duration, PICU stay, and higher mortality compared to those without VAP. Due to the complete separation of data, standard logistic regression could not be performed; however, Firth penalized logistic regression confirmed strong associations for prior antibiotic use and underlying conditions, though wide confidence intervals indicate these estimates should be interpreted cautiously. These findings suggest that VAP remains a significant burden in Pakistani PICUs. Prospective multicenter studies with larger sample sizes are needed to establish causal relationships and evaluate preventive interventions.
